# Scoliosis secondary to lumbar osteoid osteoma: A case report of delayed diagnosis and literature review

**DOI:** 10.1097/MD.0000000000005362

**Published:** 2016-11-28

**Authors:** Haiping Zhang, Xingbang Niu, Biao Wang, Simin He, Dingjun Hao

**Affiliations:** Department of Spine Surgery, Honghui Hospital, Xi’an Jiaotong University Health Science Center, Xi’an City, Shanxi Province, China.

**Keywords:** diagnosis, osteoid osteoma, scoliosis, surgery

## Abstract

**Rationale:**

Lumbar osteoid osteoma has a low incidence, which could easily lead to scoliosis.

**Patient concerns:**

Scoliosis secondary to lumbar osteoid osteoma could be easily misdiagnosed when patients do not complain of obvious symptoms.

**Diagnoses:**

We reported a case of a 9-year-old boy with back deformity that was firstly diagnosed with scoliosis at the local hospital. After prescribed with orthosis, the patient experienced aggravating pain that could not be relieved with painkillers. After he admitted to our hospital for further medical advice, he was prescribed to complete radiological examinations. Considering his radiological examination results and his medical history, correct diagnosis of lumbar osteoid osteoma was made.

**Interventions:**

Surgical intervention of posterior lesion resection was conducted after diagnosis. Intra-operative frozen pathology indicated features of osteoid osteoma. As the lesion involved inferior articular process of L5, which could cause lumbar instability after lesion resection, internal fixation was conducted at L4-S1 segment, and posterolateral bone fusion was also conducted at L5-S1 segment.

**Outcomes:**

Three months after operation, the patient showed marked improvement of scoliosis deformity and great relief of lumbar pain.

**Lessons subsections:**

Although spine osteoid osteoma is clinically rare, it shall not be overlooked when young patients present with scoliosis first. Radiological results including computed tomography and magnetic resonance imaging shall be taken carefully as reference when making diagnosis. Surgical intervention of lesion resection could well improve scoliosis and relieve lumbar pain.

## 1 Introduction

Osteoid osteoma is a benign bone lesion with high incidence at the age between 10 and 20,^[1]^ contributing to approximately 12% of benign skeletal neoplasms. It occurs more in extremities than in spine.^[2]^ Here, we reported a rare case of delayed diagnosis of scoliosis secondary to lumbar osteoid osteoma, which is difficult to diagnose when patients complain of no pain. After correcting the diagnosis, we successfully conducted posterior tumor resection, grafting bone fusion, and internal fixation. The patient reported great relief of pain as well as marked improvement in spine deformity after the operation.

## 2 Case report

### 2.1 History

**Figure 1:**
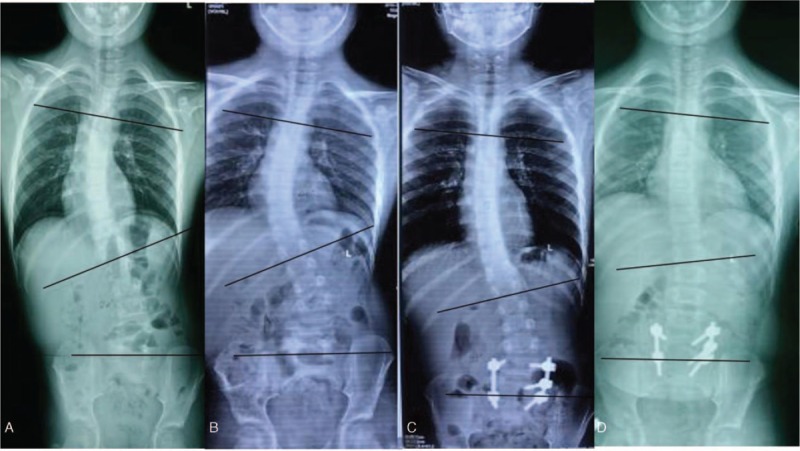
A, Spine X-ray in 2014.11: T5-T12 Cobb: 36°, L1-L5: 20°. B, Spine X-ray in 2015.12: T5-T12 Cobb: 40°, L1-L5: 27°. C, Spine X-ray 1 week after surgery: T5-T12 Cobb: 15°, L1-L5: 20°. D, Spine X-ray 3 months after surgery: T5-T12 Cobb: 10°, L1-L5: 12°.

A 9-year-old boy admitted to our hospital on December 11, 2015 complaining of scoliosis for 1 year and lumbar pain for half a year. The patient presented back deformity with asymmetrical shoulder height 1 year ago, and he admitted to the local hospital. X-ray examination was prescribed, revealing scoliosis with thoracic Cobb angle from T5 to T12 to be 36° and lumbar Cobb angle from L1 to L5 to be 20° (Fig. 1A). The patient was prescribed with orthosis, and he suffered from aggravating lumbar sacral discomfort considered to be due to scoliosis. Painkillers were later prescribed at the local hospital, which could not relieve the symptoms. As the patient experienced repeated lumbar sacral discomfort and aggravating pain, he came to our institution for further medical advice.

### 2.2 Examinations

**Figure 2:**
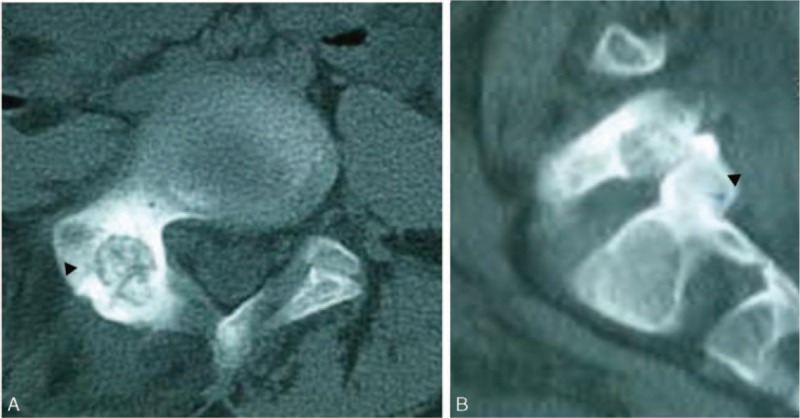
A, CT revealed a mass lesion at the right lamina of the fifth lumbar vertebra. Punctate calcification was observed within and around the lesion; reactive sclerosis change was observed around the lesions. B, Sagittal reconstruction CT showed lesions involving the right side of the fifth lumbar facet. CT = computed tomography.

**Figure 3:**
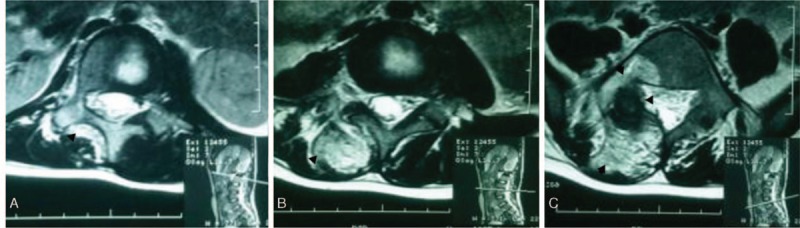
A, At the right side of L1 vertebral level, paraspinal soft tissue showed abnormal signal change. B, Paraspinal soft tissue showed abnormal signal change at the L4 vertebral level. C, T2W1 showed focal lesions with low signal and clear border at L5 level; bone marrow edema was detected at the right side of the lamina, pedicle and posterolateral part of the L5 vertebral body.

**Figure 4:**
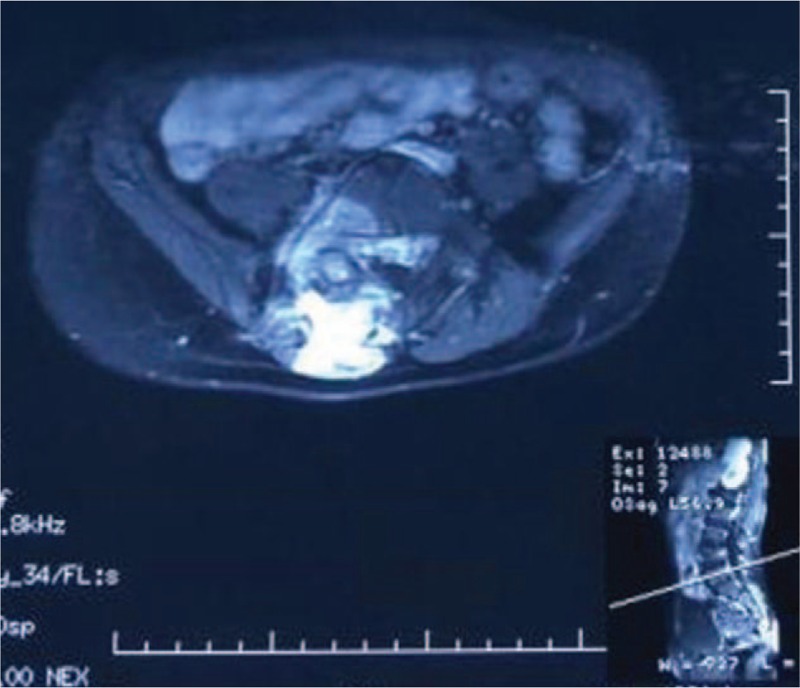
Enhanced MRI. MRI = magnetic resonance imaging.

**Figure 5:**
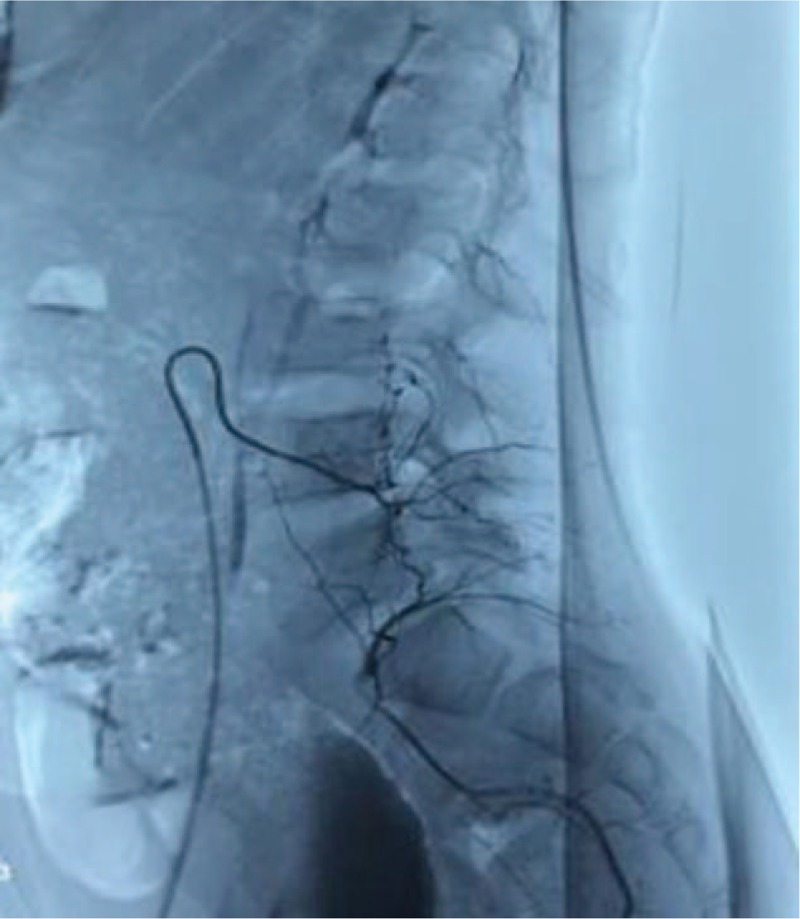
Angiography showed no abnormal blood supply.

Spinal X-ray examination (Fig. 1B) showed scoliosis with Cobb angle to be 40° from T5 to T12 and 27° from L1 to L5, combined with I degree spinal rotation according to Nash–Moe pedicle shadows method.^[3]^ Risser grade was of grade 2. Computed tomography (CT) scanning (Fig. 2) indicated focal lesion at the right vertebral laminae and inferior articular process of L5 with well-defined border and punctate calcification inside; reactive hyperplasia and sclerosis was also observed around the lesion and mixed low-density shadow was observed at the right lumbar soft tissue, indicating the possibility of infection. Lumbar magnetic resonance imaging (MRI) (Fig. 3) revealed focal lesion at L5 with clear border and low signal in T2W1; bone marrow edema could be witnessed at the posterior lateral part of right vertebral laminae, vertebral pedicle, and vertebral body; abnormal signal was detected at paravertebral soft tissue from inferior edge of L1 to S1 and enhanced MRI was applied for further investigation. The enhanced MRI (Fig. 4) showed abnormal diffused enhancement at right vertebral laminae, vertebral pedicle, the posterior lateral part of vertebral body, inside the spinal canal as well as at the right paravertebral soft tissue from inferior edge of L1 to S1. Combined with the patient's medical history, the focal lesion at L5 was considered to be osteoid osteoma. However, as the lesion of paravertebral soft tissue could not be defined, the possibility of inflammation or malignant tumor could not be excluded. As vascular thrombosis could be conducted to avoid intraoperative bleeding if rich blood supply in the soft tissue could be verified, vertebral angiography was prescribed and the result revealed no abnormal blood supply (Fig. 5).

### 2.3 Operation and pathological findings

**Figure 6:**
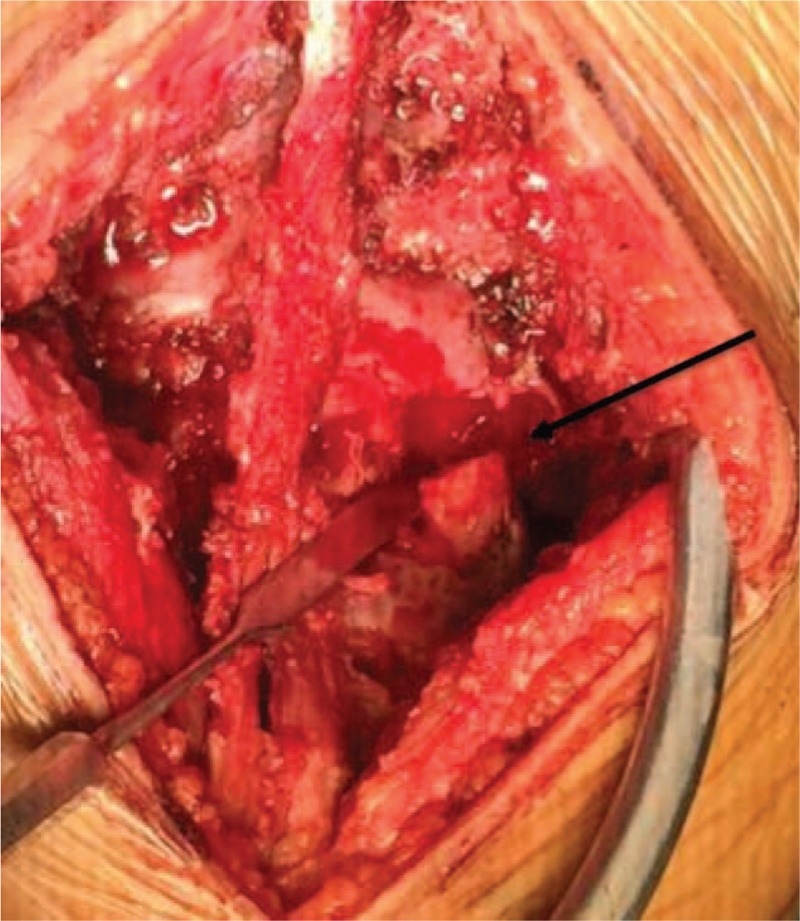
Intraoperative findings: arrow indicated the resection area, involving the right side of the fifth lumbar facet.

**Figure 7:**
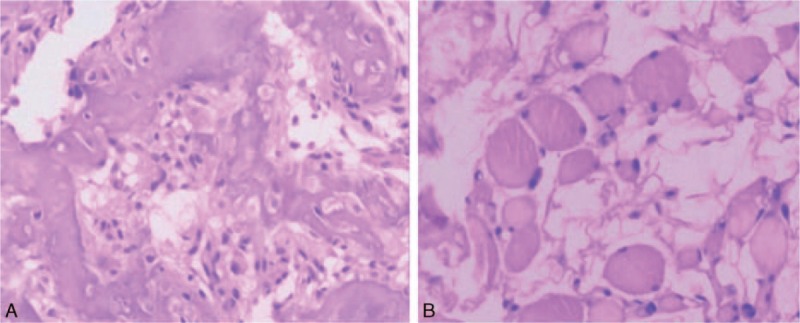
A, Bone tissue with reticular structure, composited by bone tissue, osteoid tissue, new bone tissue, and osteoblasts around the trabecular bone, confirming the diagnosis of osteoid osteoma. B, Intramuscular fat tissue hyperplasia with inflammatory changes.

After necessary preoperative examinations, posterior lesion resection was conducted (Fig. 6). The bone lesion and the adjacent soft tissue were resected for intraoperative frozen pathology. The result showed bone tissue with reticular structure, composited with bone tissue, osteoid tissue and new bone tissue. In addition, the bone trabecular was surrounded by osteoblasts, which corresponded to the features of osteoid osteoma (Fig. 7A). Furthermore, the pathology result of paravertebral soft tissue revealed fat tissue with neoplastic hyperplasia as well as inflammatory changes (Fig. 7B). Considering the radiological results as well as the pathology results, the abnormal changes of paravertebral soft tissue were considered to be inflammatory edema caused by osteoid osteoma, thus simple resection was conducted. As the lesion involved inferior articular process of L5, which could cause lumbar instability after lesion resection, internal fixation was conducted at the L4-S1 segment, and L5-S1 posterolateral bone fusion was also conducted. The patient's neurological condition remained stable during the disease course.

### 2.4 Postoperative course

**Figure 8:**
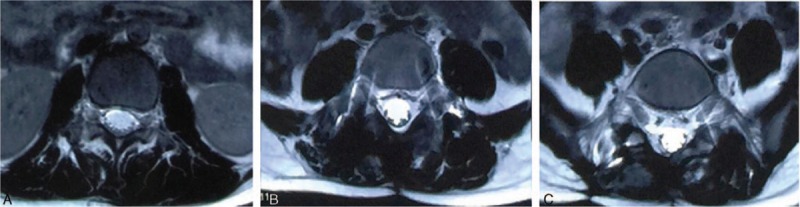
Paraspinal soft tissue showed normal signal. A, L1 level. B, L4 level. C, S1 level.

Spinal X-ray examination was conducted 1 week after operation (Fig. 1C), and the result showed obvious improvement of scoliosis deformity as the thoracic Cobb angle improved from 40°to 15°, and the lumbar Cobb angle improved from 27° to 20°. The patient was prescribed with brace therapy. At the follow-up 3 months after operation, the patient showed marked improvement of scoliosis and reported obvious relief of lumbar pain. The spinal X-ray showed that the thoracic Cobb angle improved to 10° and the lumbar Cobb angle improved to 12 ° (Fig. 1D). The MRI (Fig. 8) revealed normal signal at paravertebral soft tissue compared with the preoperative result (Fig. 3).

Our case report has received approval from Honghui hospital Institutional Review Broad (IRB).

## 3 Discussion

### 3.1 General features

Osteoid osteoma is a common benign tumor with high incidence in teenagers and with incidence of approximately 10% to 25% in spine.^[4,5]^ The predilection sites of spinal osteoid osteoma involve posterior column, especially the vertebral laminae and vertebral pedicle,^[6]^ which could easily lead to scoliosis. Patients with osteoid osteoma often come to seek medical advice with scoliosis as the primary symptom, and osteoid osteoma could easily be misdiagnosed or even never diagnosed.

### 3.2 Diagnosis

The diagnosis of osteoid osteoma is primarily based on the clinical manifestations, examinations, laboratory examinations, as well as pathological examinations. Aggravating nocturnal pain is the typical clinical manifestation of spinal osteoid osteoma.^[4,7]^ However, for cases at the early stage of osteoid osteoma, they may not present with obvious pain.^[8]^ The incidence of osteoid osteoma-related scoliosis varies from 20% to 70%,^[9–11]^ and spinal scoliosis is mainly due to chronic muscle cramps and inflammatory reactions surrounding the tumor.^[12]^ X-ray examination has its limitation in diagnosing osteoid osteoma as the tumor nest often locate at concave side of the apical segment; by contrast, radionuclide bone scanning has its advantage in locating the lesion.^[13]^ In addition, thin layer CT and reconstruction CT could also define the size, range and location of tumor nest. The MRI manifestations of osteoid osteoma could be various, for example, soft tissue mass, spinal cord edema, etc. It should be noted that osteoid osteoma often present with spinal cord edema combined with soft tissue inflammation, which could lead to misdiagnosis like inflammatory change or malignant tumor.^[14,15]^ The misdiagnosis rate is reported to be as high as 54.5%.^[9]^ Uehara et al^[1]^ reported a case of misdiagnosis of osteoid osteoma at the lamina of the 10th thoracic vertebra, which they presumed to be scoliosis and planned to perform posterior correction surgery, but as the preoperative CT examination revealed manifestations of osteoid osteoma, they corrected the diagnosis.

### 3.3 Treatment

In our case, the patient experienced marked improvement in clinical symptoms like pain and scoliosis deformity after lesion resection, internal fixation, as well as postoperative brace therapy. In addition, as the soft tissue edema in our case was mainly caused by the inflammatory factors released from the tumor due to the rupture of osteoid osteoma wall (as indicated in Fig. 2B), the edema signal disappeared 3 months after operation (as indicated in Fig. 8). Currently, surgical treatment is the most common way of treating osteoid osteoma.^[2,7]^ However, some studies suggested that osteoid osteoma has excellent self-healing property and nonsteroid anti-inflammatory drugs (NSAIDs) could well control the disease condition.^[16]^ Burn et al^[11]^ reported 4 cases of spinal osteoid osteoma presenting as pain-related scoliosis, among which 2 cases received surgical treatment and 2 cases received nonsurgical treatment with equivalent follow-up outcomes. They suggested that indications for surgical intervention included failed to control symptoms with NSAIDs, marked neurological deficits, and requirement for pathological diagnosis. As drug therapy could yield long treatment cycle and spinal osteoid osteoma often combines with spinal deformity, the long-term drug therapy will do no good to deformity correction, and we believe that surgical resection is the preferred method in alleviating clinical symptoms and correcting osteoma-related deformity. As postoperative instability was reported in 20% to 50% of the patients who underwent surgical osteoid osteoma excision,^[17]^ proper surgical method and excision range based on precise preoperative orientation and careful operation are important to maintain spinal stability.^[18]^ In terms of our case, inferior articular process of L5 was involved, which could cause spine instability after resection, thus L4-S1 internal fixation and posterolateral bone fusion were conducted. In addition, minimally invasive treatment for spine osteoid osteoma like percutaneous radiofrequency thermal ablation^[19]^ and laser thermocoagulation^[20,21]^ have been promoted recently but conflicting results have been reported as nidus at the posterior part of the spine could inevitably cause thermal damage to the nerves.^[21]^ These noninvasive treatments require further investigation for treating spinal osteoid osteoma.

Even though spinal osteoid osteoma is clinically rare, it will be taken into consideration when young patients present with scoliosis. Radiological examinations like CT and MRI will be completed to avoid misdiagnosis. Surgical resection could well improve scoliosis and relieve lumbar pain.
